# Transcriptome Analysis and In Situ Hybridization for FcaGHV1 in Feline Lymphoma

**DOI:** 10.3390/v10090464

**Published:** 2018-08-30

**Authors:** Mahdis Aghazadeh, Mang Shi, Patricia A. Pesavento, Amy C. Durham, Tamsen Polley, Shannon L. Donahoe, Ryan M. Troyer, Vanessa R. Barrs, Edward C. Holmes, Julia A. Beatty

**Affiliations:** 1 Sydney School of Veterinary Science, Faculty of Science, University of Sydney, Sydney, NSW 2006, Australia; mahdis.aghazadeh@gmail.com (M.A.); shannon.donahoe@sydney.edu.au (S.L.D.); vanessa.barrs@sydney.edu.au (V.R.B.); 2 School of Life and Environmental Sciences and Sydney Medical School, Charles Perkins Centre, University of Sydney, Sydney, NSW 2006, Australia; mang.shi@sydney.edu.au (M.S.); Edward.Holmes@sydney.edu.au (E.C.H.); 3 Marie Bashir Institute for Infectious Diseases and Biosecurity, The University of Sydney, Sydney, NSW 2006, Australia; 4 School of Veterinary Medicine, Department of Pathology, Microbiology, and Immunology, University of California at Davis, Davis, CA 95616, USA; papesavento@ucdavis.edu (P.A.P.); tmpolley@ucdavis.edu (T.P.); 5 Department of Pathobiology, University of Pennsylvania School of Veterinary Medicine, Philadelphia, PA 19104, USA; amycd@vet.upenn.edu; 6 Department of Microbiology and Immunology, Schulich School of Medicine and Dentistry, University of Western Ontario, London, ON N6A 5C1, Canada; rtroyer@uwo.ca

**Keywords:** gammaherpesvirus, immunodeficiency, virus, domestic cat, felid, lymphomagenesis, oncogenic, transcriptome, lymphoma, cancer, B-cell, T-cell

## Abstract

Lymphoma is one of the most common malignancies in domestic cats. The lymphomagenic potential of *Felis catus* gammaherpesvirus 1 (FcaGHV1), a common infection in domestic cats, is unknown. In other species, including humans, cellular transformation by gammaherpesviruses is typically mediated by viral genes expressed during latency. We analysed tumour RNA, from diffuse large B-cell lymphomas (DLBCL) appearing in cats coinfected with FcaGHV1 and feline immunodeficiency virus (FIV) (*n* = 10), by high throughput transcriptome sequencing and reverse transcription PCR. A limited repertoire of FcaGHV transcripts was identified in five tumors, including homologs of oncogenic latency-associated transcripts, latency-associated nuclear antigen (LANA, ORF73) and vFLIP (F7), lytic genes (ORF50, ORF6, ORF59, F10), and an ORF unique to FcaGHV1, F20. In situ hybridization of FIV-associated DLBCLs (*n* = 9), post-transplant lymphomas (*n* = 6) and high-grade B and T-cell intestinal lymphomas (*n* = 8) identified a single case in which FcaGHV1 nucleic acid was detectable. These results demonstrate that FcaGHV1 transcripts can be detected in some FIV-associated lymphomas, but at low copy number, precluding assessment of a potential role for FcaGHV1 in lymphomagenesis. Future investigation of the FcaGHV1 transcriptome in clinical samples might employ viral enrichment and greater sequencing depth to enhance the retrieval of viral reads. Our results suggest prioritization of a subset of intestinal T-cell tumors, large granular lymphocyte lymphoma, for study.

Felis catus gammaherpesvirus 1 (FcaGHV1; proposed species *Felid gammaherpesvirus 1*) was first reported in domestic cats in 2014 [1]. The targeted search that discovered FcaGHV1 was predicated on the hypothesis that a gammaherpesvirus would be causal in feline immunodeficiency virus (FIV)-associated lymphoma, just as the Epstein-Barr virus (EBV) and Kaposi’s sarcoma-associated herpesvirus (KSHV), are causal in 30–95% of HIV-associated lymphomas in humans [2,3]. Understanding the pathogenic potential of FcaGHV1 in cats is relevant to an expanding companion animal health market and may provide insights into human disease.

Herpesviruses alternate between lytic and latent replication cycles. During viral latency, the genome is maintained as an episome and a limited repertoire of genes is transcribed. Latency-associated transcripts collectively confer a survival advantage to maintain a viral reservoir through diverse mechanisms that promote cell growth, inhibit apoptosis and downregulate immunological targets [4]. These same genes promote cell transformation and gammaherpesvirus-associated cancers, including lymphoma. Oncogenesis occurs in a minority of gammaherpesvirus infections, often many years after the primary infection. Predisposing factors, such as immunodeficiency, are identified in some, but not all cases [5]. Although rare, gammaherpesvirus-associated cancers are frequently fatal. A full understanding of the mechanisms by which specific gammaherpesviruses transform cells is evolving, but the presence of transcriptionally active virus within cancer cells is a prerequisite to establish a direct causal relationship. In addition to established repertoires of latency-associated transcripts, an increasing number of lytic genes are implicated in tumorigenesis [6,7,8,9]. The role of gammaherpesviruses in oncogenesis can be informed from the spectrum of genes transcribed in cancer cells.

FcaGHV1 infection has been reported in cats from Australia, USA, UK, central Europe, Japan, Singapore and Brazil, with 9.6% to 23.6% testing positive for FcaGHV1 on whole blood quantitative real-time PCR (qPCR) [1,10,11,12,13,14]. One study showed that anti-FcaGHV1 antibodies identified persistent infection in 35% of cats, and the true infection prevalence may be higher [15]. So, while FcaGHV1 infection is common, any disease that it might cause is expected to be rare. Defining the pathogenicity of FcaGHV1 will rely on accumulating evidence from multiple lines of investigation.

We investigated the transcriptome of FIV-associated lymphomas with complimentary techniques, deep sequencing and reverse transcriptase (RT)-PCR, to identify FcaGHV1 transcription. In situ hybridization (ISH) was used to identify the cellular location of FcaGHV1 nucleic acid in FIV-associated lymphomas, post-transplant lymphomas and high-grade B and T-cell gastrointestinal lymphomas.

Transcriptome analysis was performed on large cell, high-grade B-cell lymphomas consistent with diffuse large B cell lymphoma (DLBCL), arising in cats infected with FIV [16]. The antemortem diagnosis of FIV was based on a positive serology result in a cat that had not been vaccinated against FIV [17]. Necropsy samples were stored at −80 °C for transcriptome analysis. Sample collection was approved by the University of Sydney Animal Ethics Committee N00/7-2013/3/6029 and 2014/626. Cases were included if they tested positive for FcaGHV1 DNA in whole blood by qPCR or tumour tissue by conventional PCR (cPCR) [1,18] (*n* = 10). Cases were excluded if they tested seropositive for the directly oncogenic gammaretrovirus feline leukemia virus (FeLV) [19]. FIV infection status was confirmed by PCR of tumour-derived DNA for FIV gag, as described previously [18]. Total RNA was extracted from frozen tumour, as described previously [20]. In the first round of NGS, libraries were prepared for cases 1, 2 and 8 using a TruSeq RNA library preparation kit (Illumina, San Diego, CA, USA). Cytoplasmic ribosomal RNA was depleted using a Ribo-Zero Gold rRNA removal kit (human/mouse/rat) (Illumina, San Diego, CA, USA). The 75 bp paired-end libraries were then run on an Illumina NextSeq platform (San Diego, CA, USA). Library preparation for a second round of RNA sequencing was performed for samples 1 to 10 (Table 1) using identical preparation except that ribosomal RNA was removed using a Ribo-Zero Gold rRNA removal kit (epidemiology) (Illumina). RNA sequencing of 100 bp paired-end libraries was performed on an Illumina HiSeq 2500 platform. To estimate the abundance of FIV and FcaGHV1, the reads from each library were mapped to the corresponding genomes using Bowtie2 software [21]. The mapping results were subsequently visualized and manually examined using the Interactive Genomic Viewer (http://software.broadinstitute.org). To confirm the mapping results, we also performed blastn [22] and diamond blast [23] analyses of reads, against the comprehensive non-redundant nucleotide (nt) and protein (nr) databases, respectively. The reads identified as FcaGHV1 transcripts were consistent with those discovered by the mapping approach. For comparison, the reads were also mapped to beta-glucuronidase (GUSB) which is stably expressed in cats [24]. Viral reads mapping to FcaGHV1 lytic gene homologs ORF50, ORF6, ORF59, F10 were recovered. ORF50 triggers reactivation from latency and inhibits apoptosis [25], F10, a KSHV K3 homolog, and downregulates MHC-I, whereas ORF59 and ORF6 encode a polymerase and a DNA binding protein, respectively. Two reads mapped to an ORF unique to FcaGHV1, F20, of unknown function [26].

RT-PCR of tumor RNA was performed as an alternative approach to identify FcaGHV1 transcripts. Total tumor RNA was prepared as before. RNA quality and purity, examined using an Agilent Bioanalyzer 2100 (Agilent Technologies, Melbourne, Australia), demonstrated RNA integrity numbers >8. Primers were designed to amplify FcaGHV1 ORF50, which had been identified on NextSeq, as well as FcaGHV1 ORF73 and F7, predicted to encode homologues of latency-associated nuclear antigen (LANA) and vFLIP, respectively, both of which are lymphomagenic in transgenic mice (Table 2) [26,27]. One-step RT-PCR was used to detect viral transcripts in low abundance (One-step Ahead RT-PCR kit; Qiagen, Hilden, Germany) using up to 1 µg of RNA as the template. Reverse transcription was carried out at 52 °C for 10 minutes. This was followed by PCR activation at 95 °C for 5 min, a 35 cycles of denaturation at 95 °C for 1 min, annealing at 56–57 °C for 15 s, extension at 72 °C for 10 s, and a final extension at 72 °C for 5 min. The identity of PCR products migrating at the expected size on gel electrophoresis was confirmed by Sanger sequencing (Macrogen sequencing, Seoul, Korea).

For ISH, formalin-fixed paraffin-embedded (FFPE) tissues from 9 of the FIV-associated lymphomas were investigated. In addition, archived diagnostic samples from post-renal transplant large B-cell lymphomas (*n* = 6) and high-grade B- and T-cell gastrointestinal lymphomas (*n* = 8) were retrieved. The causal role for gammaherpesviruses in lymphomas arising in human transplant recipients provided the rationale for investigating feline post-transplant lymphomas [28]. High grade gastrointestinal lymphomas were included because the global control of FeLV has seen these tumors become a dominant subtype and their cause is unknown [29,30]. FFPE tumour-derived DNA from post-transplant and gastrointestinal tumours was tested for FcaGHV1 by cPCR prior to ISH, as described previously [18]. An anti-sense ISH probe for FcaGHV1 ORF50 was custom-designed (Advanced Cell Diagnostics, Newark, CA, USA). This probe can detect both DNA and RNA, where the target is a double-stranded DNA virus, such as a herpesvirus. Four-micron tissue sections were prepared on charged glass slides (Superfrost Plus, Thermoscientific, Waltham, MA, USA). Sample preparation and pretreatment for the RNAscope 2.5 Assay were carried out according to the manufacturer’s instructions (Advanced Cell Diagnostics, Newark, CA, USA). Hybridized probe was identified with RNAscope® 2.5 HD Detection Reagent-Red. Samples were tested with an FcaGHV1 ORF50 probe, and a probe for bacterial gene DapB served as a negative control.

Transcriptomic analyses detected a limited repertoire of FcaGHV1 transcripts (100% match) in 5 of 10 FIV-associated lymphomas (Table 1). FcaGHV1 transcripts included homologs of KSHV latency-associated transcripts LANA and vFLIP, which induce B-cell lymphomas in mice [31,32]. LANA, KSHVs major latency protein, contributes to the maintenance of episomal DNA in proliferating cells, by tethering the episome to the host genome. The mechanisms by which LANA promotes KSHV-associated lymphomagenesis include the inhibition of tumor suppressor genes *p53* and *Rb*, and stabilization of protooncogene-encoded MYC [27]. vFLIP has anti-apoptotic effects mediated by preventing the activation of the protease, caspase 8 [27]. Although only 2 to 4 reads per FcaGHV1 transcript were detected, we believe that the transcripts can be considered genuine as they were identified using multiple approaches. In addition, we initially discovered a novel domestic cat hepadnavirus from 8 transcripts in case 6, which was later confirmed by PCR in a larger number of animals [20].

Metatranscriptomics of clinical samples can reveal the pattern of viral gene expression within cancer cells, without bias introduced by viral enrichment. Nonetheless, the direct investigation of clinical samples presents several challenges. Firstly, host non-rRNA reads are expected to dwarf those of a latent virus. The very low abundance of transcripts retrieved, suggests that the sensitivity of these methods is close to the lower limit of detection for FcaGHV1 transcripts in clinical specimens. Secondly, tumors are heterogeneous containing inflammation and necrosis alongside cancer cells, and cells undergoing lytic as well as latent virus replication. This can contribute to inconsistency in results as a consequence of sampling different regions of the same tumor. Future investigations of the FcaGHV1 transcription profile in clinical specimens, invoking deeper sequencing, library baiting, and absolute quantitation are expected to yield valuable information.

One post-transplant lymphoma and one gastrointestinal lymphoma tested positive for FcaGHV1 by cPCR. From 23 tumors tested by ISH, a single gastrointestinal lymphoma yielded a positive result. This tumor, a high-grade, T-cell lymphoma, subtyped as a large granular lymphocyte lymphoma, was the sample that tested positive for FcaGHV1 by cPCR. A positive intranuclear signal was identified in >90% of neoplastic lymphocytes. This result has been repeatable in two laboratories (PP and JB) (Figure 1). An identical pattern of staining was obtained when this tumor was tested by ISH for FcaGHV1 ORF73 and with a combined F7, F10, F20 probe. In contrast, the other 22 lymphomas were consistently negative for FcaGHV1 on ISH, and the unrelated control probe gave a negative result in all 23 samples with ISH. In the single ISH positive tumor, the consistent intranuclear signal and the absence of a cytoplasmic signal with any FcaGHV1 ISH probe, supports that episomal FcaGHV1 DNA is maintained in the majority of neoplastic lymphocytes in this intestinal T-cell, large granular lymphocyte lymphoma. Further studies are required to understand whether the continued presence of the virus is required to maintain tumor phenotype, as it is with other GHV-associated cancers [33]. Of note here, the published FcaGHV1 genome sequence was obtained from a lymph node, selected for its high FcaCaGHV1 load of 48 FcaGHV1 genomes per cell, from a case of high-grade, T-cell intestinal lymphoma [26]. 

In summary, FcaGHV1 transcripts can be detected in some FIV-associated lymphomas, but at low copy number which precludes assessment of a potential role for FcaGHV1 in lymphomagenesis. Our results identify a subset of intestinal T-cell tumors, large granular lymphocyte lymphoma that can be targeted in future investigations of the pathogenic potential of FcaGHV1.

## Figures and Tables

**Figure 1:**
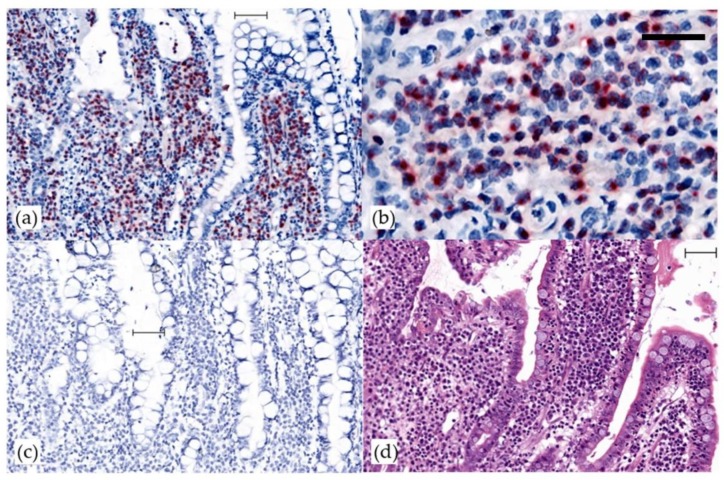
Detection of FcaGHV1 by in situ hybridization in an intestinal large, granular lymphocyte lymphoma. In situ hybridization for FcaGHV1 ORF 50. Bars 50 micrometers. (**a**) A strong intranuclear signal (red) labels >90% of infiltrating neoplastic lymphocytes that are densely packed within intestinal villi. Counterstain, hematoxylin; (**b**) high power image to show nuclear staining of neoplastic lymphocytes; (**c**) no staining with negative control probe DapB (**d**) hematoxylin and eosin

**Table 1:** Summary of transcriptome analyses of feline immunodeficiency virus associated lymphoma from FcaGHV1 infected cats.

Cases	NextSeq FcaGHV1 Reads	HiSeq Number of Reads				RT-PCR		
		Total	GUSB	FIV	FcaGHV1	FcaGHV1 ORF50	FcaGHV1 ORF73	FcaGHV1 F7
1	Neg	294,779,800	4149	1023	0	Neg	Neg	Neg
2	4 (ORF 50)	338,384,008	8070	2959	0	POS	POS	POS
3	-	288,176,064	4152	48,270	0	Neg	Neg	Neg
4	-	317,655,184	6526	32	0	POS	Neg	POS
5	-	300,408,704	1608	4090	0	Neg	Neg	Neg
6 ^1^	-	321,297,408	12,866	1636	2 (ORF59)	Neg	Neg	POS
7	-	337,067,856	21,636	100	0	Neg	Neg	Neg
8	Neg	328,445,328	5103	2207	0	POS	Neg	POS
9	-	313,060,728	1410	141	2 (F10) 2 (F20) 2 (ORF6)	Neg	Neg	Neg
10	-	239,825,576	1973	11	0	Neg	Neg	Neg

Cases are large cell, high-grade B-cell lymphomas from feline immunodeficiency viruses (FIV) and *Felis catus* gammahepresvirus 1 (FcaGHV1) infected cats. Numbers represent reads obtained from high throughput sequencing of tumor RNA (NextSeq and/or HiSeq). Total, beta-glucuronidase (GUSB), and viral reads are presented. For FcaGHV1, the region to which reads map is shown in brackets. Results of RT-PCR of tumor RNA for FcaGHV1 ORF50, ORF 73 and F7 are indicated as positive (POS) or negative (NEG). The identify of amplicons obtained by RT-PCR was confirmed by sequencing. ^1^ A novel hepadnavirus discovered in this case is reported elsewhere [20].

**Table 2:** Oligonucleotides used to detect *Felis catus* gammaherpesvirus 1 transcripts in feline immunodeficiency virus -associated lymphoma by RT-PCR.

Oligonucleotide	Sequence	Size (bp)	Tm (°C)
ORF50-F1	5′-CCCAGGGCTTTTTGTGTGGA-3′	301	57
ORF50-R1	5′-GGGCTTGACTCATAAGGGCA-3′		
ORF73-F2	5′-CAACTGGGCATTGGCATAC-3′	110	57
ORF73-R2	5′-CCTTAGTTCACCCAACTTGTGC-3′		
F7-F	5′-ACTCTGTGTCTGGGAATGTGAC-3′	153	56
F7-R	5′-TGGCTTGTGTATATGGCCAGC-3′		
